# A Parent-Implemented Language Intervention for Late Talkers: An Exploratory Study on Low-Risk Preterm and Full-Term Children

**DOI:** 10.3390/ijerph17239123

**Published:** 2020-12-07

**Authors:** Mariagrazia Zuccarini, Chiara Suttora, Arianna Bello, Arianna Aceti, Luigi Corvaglia, Maria Cristina Caselli, Annalisa Guarini, Alessandra Sansavini

**Affiliations:** 1 Department of Psychology “Renzo Canestrari”, University of Bologna, 40127 Bologna, Italy; mariagrazia.zuccarini@unibo.it (M.Z.); chiara.suttora@unibo.it (C.S.); 2 Department of Education, Roma Tre University, 00154 Rome, Italy; arianna.bello@uniroma3.it; 3 Neonatology and Neonatal Intensive Care Unit, S. Orsola-Malpighi Hospital, 40138 Bologna, Italy; arianna.aceti2@unibo.it (A.A.); luigi.corvaglia@unibo.it (L.C.); 4 Department of Medical and Surgery Sciences, University of Bologna, 40138 Bologna, Italy; 5 Institute of Cognitive Sciences and Technologies, CNR, 00185 Rome, Italy; cristina.caselli@istc.cnr.it

**Keywords:** parent-implemented intervention, expressive language delay, low-risk preterm children, late talkers, MB-CDI

## Abstract

Parent-implemented language interventions have been used for children with expressive language delays, but no study has yet been carried out using this intervention for low-risk preterm children. The current study examined the effect of a parent-implemented dialogic book reading intervention, determining also whether the intervention differently impacted low-risk preterm and full-term children. Fifty 31-month-old late talkers with their parents participated; 27 late talkers constituted the intervention group, and 23 constituted the control group. The overall results indicated that more children in the intervention group showed partial or full recovery of their lexical expressive delay and acquired the ability to produce complete sentences relative to the control group. Concerning full-term late talkers, those in the intervention group showed a higher daily growth rate of total words, nouns, function words, and complete sentences, and more children began to produce complete sentences relative to those in the control group. Concerning low-risk preterm late talkers, children in the intervention group increased their ability to produce complete sentences more than those in the control group. We conclude that a parent-focused intervention may be an effective, ecological, and cost-effective program for improving expressive lexical and syntactic skills of full-term and low-risk preterm late talkers, calling for further studies in late talkers with biological vulnerabilities.

## 1. Introduction

Several studies have highlighted that 9% to 20% of 2- to 3-year-old children are late talkers [1,2,3,4]. Conventional criteria for identifying late talkers include an expressive vocabulary size at or below the 10th percentile assessed by parental questionnaires, such as the MacArthur–Bates Communicative Development Inventories (MB-CDI) [5,6,7,8] and/or absence of word combinations between 24 and 30 months of age [4,9]. A further conventional criterion for being classified as late talker is the absence of neurological and developmental disorders, intellectual disabilities, hearing impairments, and socio-emotional deficits [6,10,11,12].

The prevalence of late talkers has been found to increase in children with vulnerabilities associated to specific biological conditions, such as preterm birth [13]. Although literature findings are still controversial, rates of language delay in preterm children comprise between 24% and 32% in children with very low gestational age (<32 weeks) [13,14]. Less concordant findings were found by studies including low-risk preterm children characterized by a lower immaturity and a lower incidence of severe perinatal complications [15,16].

Concerning the developmental outcomes of late talkers in preschool years, longitudinal studies revealed that many of them catch up to their typically developing peers within 4 years of age [5,17]. However, 6% to 44% of late talkers have persistent language impairments, which may be associated with social and behavioral problems [18,19]. With regard to school-age outcomes, available data from small sample longitudinal studies point out that although most of these children score in the average range, they continue to exhibit weaker language, verbal memory, and reading skills compared to their typically developing peers [12]. In light of this evidence, interventions with late talkers appear relevant to reduce the impact that early language difficulties may have on later development [20].

### 1.1. Parent-Implemented Language Interventions in Late Talkers

Direct and indirect interventions have been proposed for late talkers [21,22]. Direct interventions focus on individual or group treatment of child speech and linguistic skills delivered by a speech language therapist, whereas indirect interventions foster child verbal skills by providing him/her with a facilitating and stimulating communicative environment. In indirect interventions, a specialist trains parents on how to promote positive and affective interactions with their child in daily naturalistic contexts [21]. Even if the intensity, duration, and frequency of intervention may vary among parent-based programs, their key concepts are basically very similar [20,23]. Specifically, they aimed at improving parents’ responsiveness, communicative strategies, such as expanding children’s utterances, and their ability to provide a simplified and contingent input that may increase late talkers’ opportunities to learn from their linguistic environment [20,23,24]. As pointed out by a systematic review [22], indirect interventions by parents with children aged 24 to 70 months are considered effective as direct treatments in promoting expressive vocabulary and the mean length of utterance, when these approaches are comparable in terms of intensity and dosage. Another meta-analysis [23] made similar conclusions, observing an overall lack of differences, in terms of effect sizes, in the impact of parent- and therapist-implemented interventions for language outcomes. Thus, parent-implemented programs may represent a more cost-affordable and recommended choice than one-to-one therapy for very young children [22,25].

Furthermore, indirect interventions should be preferred to the “wait and see” approach that is still widely employed in several countries [26]. As suggested by some reviews and meta-analyses [20,23], parent-implemented interventions are more effective than non-intervention in promoting language skills of children with early language difficulties. These meta-analyses [20,23] examined the outcomes of more than 20 studies, most of which were randomized control trials of parent-implemented interventions for children aged from 2 to 5 years. In particular, parent-implemented interventions improved child expressive vocabulary, albeit with no significant effects reported on child receptive abilities [20].

Several randomized controlled trial studies have been conducted in order to validate the impact of parent-implemented language intervention on late talkers, with overall positive findings. Children in the intervention group showed significantly larger expressive vocabularies and improved expressive syntactic skills, as assessed through parent-report questionnaires, compared to children in non-intervention control groups [24,25]. Interestingly, the studies used various types of intervention, for example, focused stimulation and dialogic book reading. The former trains parents to repeatedly present selected target words in the context of play routines, while the latter encourages adults to stimulate conversational exchanges in the context of book sharing. Both these types of intervention were proved to be effective in promoting child expressive language development [24,25], indicating that both dialogic book reading and play-based interventions have significant effects on child expressive vocabulary [20].

### 1.2. Parent-Implemented Language Interventions in Other Populations with Language Delay

The studies described in the previous paragraph included late talkers exhibiting expressive delay with adequate cognitive skills. Parent-implemented language interventions have also been proposed in other populations characterized by language delay, such as children with autism spectrum disorder and/or intellectual disabilities, showing contrasting results [23,27,28]. A meta-analysis and a review showed that parent-implemented language interventions foster child expressive vocabulary in these populations, although intervention appeared less effective for children at risk or with autism spectrum disorder [20,27]. By contrast, another review indicated that interventions had positive effects only on specific aspects of child–adult interaction, such as turn-taking, and on specific aspects of child language development, such as lexical diversity or mean length of utterance (MLU), but not on child expressive language vocabulary [28]. Again, these mixed findings may be due to the sample selection criteria and methodological differences of the interventions.

At present, no study has investigated the effect of parent-based language interventions with preterm children, despite the fact that this population has been widely recognized as at risk for language delay [13]. A study [29] controlled whether risk at birth (e.g., defined by admission to the neonatal intensive care unit) predicted language growth in a sample of children with language delay participating in a parent-implemented intervention, but no significant effect of this perinatal risk was observed. Therefore, evidence is needed about the effects of parent-implemented language interventions in populations at risk for language delay, such as preterm children. 

### 1.3. Objectives of the Study

We conducted the present study with two main objectives. First, this study analyzed the effect of a parent-implemented intervention in a sample of Italian late talkers comparing their outcomes to a non-treated control group. In particular, we wanted to examine the effects of the intervention on child expressive vocabulary and early expressive syntactic skills as assessed with a parental questionnaire. This study used a dialogic book reading intervention program developed for late talkers by Girolametto et al. [30]. Evidence from a pilot study using this program on a small sample of Italian late talkers [31] suggested that it may be an innovative, manageable, and cost-effective parent-implemented language intervention for late talkers. The pilot study [31], conducted with parents of eleven late talkers aged 28 to 30 months, revealed a significant increase in child expressive vocabulary, assessed through a questionnaire, a direct task, and the analysis of spontaneous language production. These findings are promising, but further research using a non-intervention control group is required to verify its efficacy. According to the results of the pilot study, we thus expected to find a higher rate of growth in the expressive lexical and syntactic skills of late talkers participating in the intervention compared to those in the control group.

The second objective was to explore whether this parent-implemented intervention differentially impacted low-risk preterm versus full-term late talkers. As this was the first time that this dialogic book reading program was used in late talkers born preterm, this second objective was considered explorative in its nature.

## 2. Materials and Methods 

### 2.1. Participants

Parents of 62 late-talking children, who were assessed for language delay at around 31 months of age (*M* = 31.20 months, *SD* = 1.04) at the Developmental Psychology Lab at the University of Bologna, were invited to participate in this study. Measures of language delay assessment served as pre-intervention assessment. The intervention lasted approximately 2 months, and post-intervention assessment occurred when the children were about 37 months of age (*M* = 36.97 months, *SD* = 1.23).

The sample included 27 low-risk preterm children, born before 37 weeks of gestational age at Sant’Orsola-Malpighi Hospital of the University of Bologna and living in the metropolitan area of Bologna, and 35 full-term children, born in the same hospital and living in the same metropolitan area with similar socio-demographic characteristics. Inclusion criteria included monolingual (Italian) or mainly exposed to the Italian language since birth. Exclusion criteria were the presence of major cerebral damage and/or congenital malformations, visual, hearing, or motor impairments, severe cognitive deficits (IQ < 70), and severe neonatal complications. None of the children had participated in previous language interventions.

Parents of 59 out of 62 late talkers accepted to participate in the study. Among them, two did not fill out the pre-intervention assessment questionnaire and were thus excluded; therefore, parents of 57 late talkers were included in the study. Using a convenience sample methodology, parents were asked whether they were available to participate in the intervention based on their time schedule, family organization, and interest in the project. Thirty parents accepted to participate in the intervention (intervention group), whereas 27 parents did not (control group) for several reasons (i.e., for time-scheduling problems, *n* = 25, or because not interested in the intervention, *n* = 2), but they agreed on participating in the post-intervention assessment. In the intervention group, 3 children were excluded because of family difficulties in scheduling the post-intervention assessments by 37 months of age. Thus, the final intervention sample consisted of parents of 27 children. Concerning the control group, 4 children missed the post-intervention assessment because of family or medical issues (*n* = 2), the initiation of individual treatment (*n* = 1), or family moving to another city (*n* = 1). Thus, the final control sample consisted of parents of 23 children.

**Figure 1:**
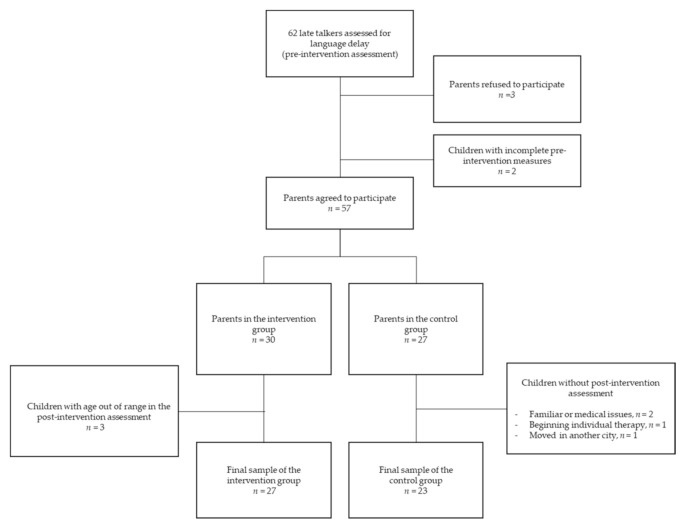
Flow diagram of late talkers whose parents participated in the study.

The flow diagram of participants was described in Figure 1. 

The biological and sociodemographic characteristics of children and parents belonging to the intervention and control groups are presented in Table 1. Participants from the two groups were similar in most sociodemographic characteristics except for mothers’ and fathers’ ages, which were significantly higher in the intervention group. Children’s ages at the pre and post-intervention assessments and the interval between pre- and post-assessments are also reported in Table 1, revealing no significant differences. Age corrected for gestational age was used for low-risk preterm children considering their level of neurobiological maturation as done in previous studies [15,32].

Intervention and control groups significantly differed in the distribution of low-risk preterm and full-term children: the intervention group included parents of 17 low-risk preterm children and 10 full-term children, whereas the control group consisted of parents of 6 low-risk preterm children and 17 full-term children, χ^2^(1, 50) = 6.80, *p* = 0.009. 

We also compared biological and sociodemographic characteristics of the intervention and control groups within low-risk preterm and full-term samples, according to the second objective (Table 2).

Within the low-risk preterm group, maternal age and attendance at nursery school were higher in the intervention than in the control group (Table 2). In addition, children participating in the intervention group were slightly younger than those in the control group at the pre-intervention assessment but not at the post-intervention assessment. The interval between pre and post- intervention did not differ among groups. Perinatal and clinical characteristics of low-risk preterm children are reported in the Appendix A. 

Within the full-term group, no difference was highlighted between the intervention and control group for children’s and parents’ characteristics (Table 2) nor in children’s age at pre-intervention and post-intervention assessment.

### 2.2. Procedure and Study Design

At the pre-intervention assessment, parents were asked to report on their child expressive lexical and syntactic skills by filling out the Italian version of the MacArthur Bates Communicative Development Inventories (CDI), Words and Sentences Complete Form [33]. Cognitive skills of children were assessed with the use of the Bayley Scales of Infant and Toddler Development, Third Edition (BSID-III) [34,35] (see Paragraph 2.3 for tool description).

After the pre-intervention assessment, parents who accepted to participate in the intervention attended six sessions, each lasting approximately 2 h (as explained in Paragraph 2.4). 

In order to evaluate the effectiveness of the intervention, a pre–post-intervention assessment design was used, with a post-intervention assessment occurring at about 6 months after the pre-intervention assessment. Therefore, at the post-intervention assessment, parents were asked to fill out the same questionnaire used in the pre-intervention assessment.

### 2.3. Tools

Language skills were assessed through the Italian version of the MacArthur Bates Communicative Development Inventories (MB-CDI), Words and Sentences Complete Form [33]. The MB-CDI Complete form is a reliable and valid tool for evaluating expressive lexical and syntactic development of toddlers [33] and has been used in several parent-implemented language intervention studies involving late talkers [36]. The Italian version has been validated on 752 Italian children aged 18–36 months [33].

Children’s expressive vocabulary was assessed through the MB-CDI checklist of 670 words, which were divided into 23 semantic categories. The macro-categories were the following: social words (onomatopoeia, routine words and people names), nouns (animals, vehicles, toys, food and drink, clothing, body parts, small household items, furniture and rooms, outside things, places to go), predicates (verbs and adjectives), function words (pronouns, question words, prepositions and locations, quantifiers and articles, connecting words), adverbs (words about time and location), and modal verbs. Parents were requested to indicate if their child spontaneously produced the words in the checklist. A score of 1 was given for each item checked. For the purposes of the present study, the total number of words produced was computed to obtain an overall score of expressive vocabulary. A total score was also calculated for each macro-category by summing the items corresponding to social words, nouns, predicates, and function words. In addition, a measure of child progress in expressive vocabulary in relation to MB-CDI Italian standardized norms was computed. Using the Italian MB-CDI normative values [33], child total word production scores at pre-intervention and post-intervention assessment were transformed into percentile values, and children were categorized into three different percentile ranks: ≤5th percentile, between the 6th and the 10th percentile, and >10th percentile. Based on their improvement, children were categorized into two groups. The first group named “not recovering” included late-talkers whose expressive vocabulary showed stability or decrease in percentile ranking (i.e., children ≤ 5th percentile or between the 6th and the 10th percentile at the pre-intervention and post-intervention assessment). The second group named “partially or fully recovering” included children who progressed in percentile ranking showing partial or complete recovery from their expressive lexical delay (i.e., children ≤ 5th percentile at the pre-intervention assessment that moved to the 6th–10th percentile or above the 10th at the post-intervention assessment; and children in the 6th–10th percentile at the pre-intervention assessment that moved above the 10th percentile at the post-intervention assessment).

Expressive syntactic skills were assessed through a checklist of 37 sentence pairs. Parents were asked to indicate for each pair of sentences which one better reflected the actual complexity of child spontaneous sentence production. Within each pair, the first sentence was presented in a telegraphic style (incomplete sentence lacking function words), whereas the second in a complete style including function words (complete sentence). A score of 1 was given for each item checked. To assess child sentence production, the number of incomplete, complete, and total sentences was computed. In addition, as the use of complete sentences is a clear index of advanced grammatical skills, children were classified into three categories depending on their progresses in the production of complete sentences: “stable incomplete”, i.e., children who did not produce complete sentences at pre- and post-intervention assessment; “emergent complete”, i.e., children who did not produce complete sentence at pre-intervention assessment and produced at least one complete sentence at post-intervention assessment; “stable complete”, i.e., children who produced at least one complete sentence both at pre- and post-intervention assessment.

To control for interindividual differences in the time interval between the pre and post-intervention assessment, for each MB-CDI variable, a measure of daily growth rate was computed by subtracting the number of items at the pre-intervention to those obtained at the post-intervention and further dividing this value for the interval between the two assessments expressed in days. Thus, the daily growth rate was computed for the following variables: total words, social words, nouns, predicates, function words, complete sentences and total sentences.

Cognitive skills of children were assessed with the use of the Bayley Scales of Infant and Toddler Development, Third Edition [34,35], computing for each child a cognitive composite score (*M* = 100, *SD* = 15). The Bayley-III Scales are a valid and widely used tool for research and clinical practice with satisfactory reliability and validity values [34]. 

### 2.4. Parent-Administered Intervention Program 

A dialogic book reading program was used. The program, called “Oltre il Libro”, is a 2-month structured and manualized parent-coaching intervention for late talkers aged 2–3 years [30]. The program was directed at small groups of parents (4–6 people) and consisted of 6 training sessions of about 2 h each and 2 video-feedback sessions.

The program is based on the interactive model of language intervention [30]. According to this program, parents were trained to support their child language development using a set of strategies consisting in open-ended questions, expansions of their child’s verbal utterances, and frequent adjustments of the input to match their child’s language abilities. The context in which these strategies were applied was dialogic book reading, which is a practice in which children and adults share an active conversation through “reading” an illustrated book together [37]. Dialogic book reading is often used in parent-implemented language intervention [20] because it has proven to be effective in enhancing child language growth not only in typically developing children [38], but also in toddlers and preschoolers with expressive language delay [25,36,39,40]. The dialogic book reading program used in this study included an additional strategy called focused stimulation. That is, parents were trained to use a set of five preselected target words, already understood by the child, at least three times within a brief conversational exchange. These words were inserted into the appropriate page of the books using sticky notes.

The six sessions of the dialogic book reading intervention specifically aimed at coaching parents to (a) choose books for their child, according to his/her interests and linguistic skills (*Session 1*); (b) use dialogic book reading strategies (i.e., asking open questions, attending to the child’s verbal and non-verbal responses, imitating the child) (*Session 2*); (c) choose five target words (i.e., understood but not yet produced words, motivating the child, simple in their phonetic structure, including at least one verb) and use focused stimulation on these target words (*Session 3*); expand child utterances (*Session 4*); (d) generalize the use of target words in the home context (*Session 5*); and (e) use technology (e.g., e-readers) for promoting child language (*Session 6*).

Parents attended on average 85% of the intervention’s group sessions, i.e., about 5 out of 6 sessions. In the few cases in which parents missed a group session, they were updated individually about the missed session’s contents through a written brochure sent via e-mail and subsequently contacted by phone for further explanation. Thus, all parents, even when they missed a group session, were able to go on in implementing the intervention program.

At the end of each session, parents were given a diary to be returned at the subsequent session. They were asked to fill it out daily, reporting how long they read with their child and which target words they used. Parents returned on average 81% of the diaries, i.e., about 4 out of 5 diaries. 

In addition to the weekly diary, parents were asked to video record a few minutes of a dialogic book-reading episode with their child at home on two occasions, specifically after the third and the fifth session. Then, the videos were examined by the training conductor, and parents were given feedback on their strategies’ use. Out of 27 parents, 17 parents (63%) returned the two required videos, 7 parents (26%) returned one video, and 3 parents (11%) did not return any video because of personal or technical issues.

Furthermore, at the end of each session, parents were asked to express, anonymously, their satisfaction with the contents, organization, and methods of the intervention through three five-point Likert scale questions (1 = extremely useful; 2 = very useful; 3 = useful; 4 = quite useful; 5 = not useful). All parents expressed their satisfaction at least one time for each of the three questions. The mean rating of parental responses across the six group sessions about the intervention contents (*M* = 1.58, *SD* = 0.20), organization (*M* = 1.62, *SD* = 0.20), and methods (*M* = 1.50, *SD* = 0.19) revealed that parents judged the intervention as extremely useful or very useful.

### 2.5. Ethics

The study met ethical guidelines for human subject protections, including adherence to the legal requirements of Italy, and it received formal approval from the Bologna Health Authority’s Independent Ethics Committee (numbers of formal approval documents: EM 194/2017/U_ and EM 193–2018_ 76/2013/U/Sper/AOUBo). All parents gave informed written consent for study participation, data analysis, and data publication. No incentives or benefits were provided to participants.

### 2.6. Statistical Analysis

All analyses were performed using IBM SPSS Statistic 25. The statistical tests were bilateral with a 0.05 alpha level of significance. The Kolmogorov–Smirnov test was used to check data for normality of distribution. Three MB-CDI variables (i.e., daily growth rate of complete sentences, total sentences, and function words) were not normally distributed (*p* < 0.005), whereas the remaining variables (i.e., daily growth rate of total words, social words, nouns, and predicates) were normally distributed. To deal with non-normal data, we opted for non-parametric statistics.

Before addressing the study questions, children’s cognitive and linguistic skills at pre-intervention assessment were compared between the intervention and control groups. The same analyses were performed separately for low-risk preterm and full-term children to assess whether differences in the pre-intervention assessment existed between children whose parents participated in the intervention versus those whose parents did not.

Concerning the first objective of the study, a chi-squared test was performed to determine whether children in the intervention group versus children in the control group showed an improvement in their expressive vocabularies according to the percentile ranking based on the normative data of the MB-CDI Italian version. The analysis compared the number of children “not recovering” versus the number of children “partially or fully recovering” their expressive lexical delay between the intervention and control group from pre- to post-intervention assessment. A chi-squared analysis was also used to verify improvements in the children’s production of complete sentences. Specifically, the test compared children not producing any complete sentence (“stable incomplete”), children with emerging complete sentence production (“emergent complete”), and children stable in their use of complete sentences (“stable complete”) from pre- to post-intervention assessment in the intervention and control group. Then, a series of Mann–Whitney tests were used to examine whether children in the intervention group showed higher rates of daily increase in their expressive vocabulary (total words, social words, nouns, predicates, function words) and expressive syntactic skills (complete sentences and total sentences) as assessed by the MB-CDI.

To address the second objective, further analyses were performed on the same set of variables, considering the low-risk preterm and full-term groups of children separately in order to explore whether the intervention had a different effect depending on childbirth condition. Fisher’s exact test was used wherever appropriate.

## 3. Results

### 3.1. Pre-Intervention Assessment

Considering the entire sample, no significant differences were observed in cognitive and language skills between children in the intervention and control groups at the pre-intervention assessment (Table 3). The only exception was for the use of social words, which was significantly higher in the intervention group.

Within the low-risk preterm group, no significant differences were found in cognitive and language skills between children in the intervention and control groups at the pre-intervention assessment (Table 3). In contrast, within the full-term group, children in the intervention group used significantly more social words with respect to the control group (Table 3).

### 3.2. Effects of the Parent-Implemented Intervention on Late Talkers’ Expressive Lexical and Syntactic Skills

**Figure 2:**
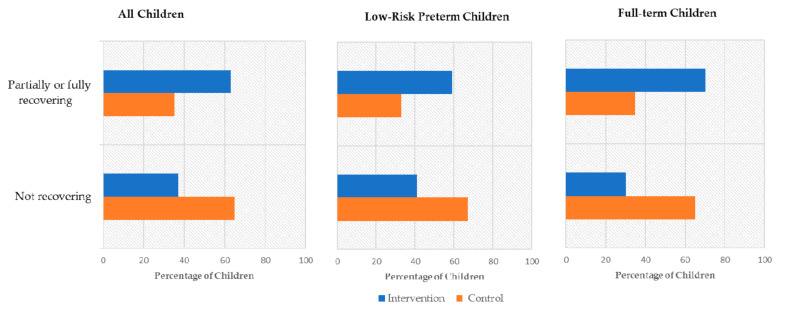
Percentage of children “partially or fully recovering” and “not recovering” expressive lexical delay from pre- to post-intervention assessment in the intervention and control group considering the whole sample and the low-risk preterm and full-term samples.

According to the first objective of the study, we analyzed whether the intervention improved the language of children in the intervention group relative to the control group (see Figure 2). In the pre-intervention assessment, 72% of children (*n* = 36) fell below the 5th percentile and the remaining 28% (*n* = 41) fell below the 10th. In the post-intervention assessment, half of the children (*n* = 25) were still showing a vocabulary size below the 5th percentile, another 10% of children were below the 10th percentile (*n* = 5), and 40% of children (*n* = 20) had values above the 10th percentile. In light of these data, children were further categorized into “not recovering” and “partially or fully recovering” groups as previously described (see Section 2.3 and Table 4). The results of the Chi-squared analysis revealed that the number of children partially or fully recovering expressive lexicon was significantly higher in the intervention group than in the control group (Table 4, Figure 2). A similar analysis was conducted to verify whether children in the intervention group showed a significant increase in the use of complete sentences when compared with children in the control group. In the pre-intervention assessment, only 18% (*n* = 9) of children exhibited the ability to produce complete sentences, whereas at post-intervention, 62% (*n* = 31) of children showed this ability. By further classifying children in “stable incomplete”, “emergent complete”, and “stable complete” (see Table 4), results showed that 63% (*n* = 17) of children in the intervention group acquired the ability to produce complete sentences versus 22% (*n* = 5) of control children. The results of the Chi-squared analysis revealed that the number of “emergent complete” children was significantly higher in the intervention group than in the control group (Table 4).

To further address our first objective, Table 5 presents descriptive statistics and results of between group comparisons for the measures of daily growth rate of word production, considering total words and semantic categories (social words, nouns, predicates, and function words), complete sentences and total sentences. Results revealed that children in the intervention group did not show significantly higher growth rates in their lexical and expressive syntactic skills when compared to their peers in the control group.

### 3.3. Effects of the Parent-Implemented Intervention on Low-Risk Preterm and Full-Term Late Talkers’ Expressive Lexical and Syntactic Skills 

The analysis of the distribution of “not recovering” children versus “partially or fully recovering” children was also conducted within the low-risk preterm and full-term groups in the intervention versus the control group (see Table 4). Both in low-risk preterm and full-term children, results highlighted the lack of significant differences in recovery from expressive lexical delay from pre- to post-intervention assessment.

Concerning the production of complete sentences (see Table 4), the statistical analyses performed showed significant effects of the intervention in both samples. In the low-risk preterm group, 53% (*n* = 9) of children participating in the intervention acquired the ability to produce complete sentences from the pre- to post-intervention assessment, whereas none of the low-risk preterm children in the control group acquired this new ability. Similarly, 80% (*n* = 8) of full-term children receiving the intervention showed an emerging use of complete sentences at the post-intervention assessment versus only 30% (*n* = 5) of the control children.

In relation to the effect of the intervention on the daily growth rate of expressive lexical and syntactic skills, no difference was highlighted between children in the intervention and the control group as regards low-risk preterm children (Table 5). By contrast, addressing full-term children, participants in the intervention group scored significantly higher than children in the control group in the daily growth rate of total words, nouns, function words, and complete sentences. Growth rates were doubled or tripled in full-term late talkers participating in the intervention versus those whose parents did not take part in it.

## 4. Discussion

The present study aimed at evaluating the effectiveness of a parent-implemented language intervention using dialogic book reading with a sample of late talkers. In addition, we explored whether this parent-implemented intervention had a differential impact on children with vulnerabilities associated to specific biological conditions, such as preterm birth, by analyzing its impact on low-risk preterm and full-term late talkers.

The findings revealed that considering the total sample, a significantly higher rate of late talkers in the intervention group showed partial or total recovery from expressive lexical delay as well as an increase in production of complete sentences compared to children in the control group. Interestingly, when we analyzed the effects of the intervention on low-risk preterm and full-term late talkers separately, we obtained differentiated results, indicating that late talkers with no additional risk factor fared better. In both low-risk preterm and full-term late talkers, a significant higher rate of children produced complete sentences after treatment than children in the non-treatment group. In addition, full-term late talkers in the intervention group achieved a significantly higher daily growth rate in total words, nouns, function words, and complete sentences compared to those in the control group, whereas this finding was not replicated for the low-risk preterm group.

The current study enriched the literature concerning the impact of parent-implemented programs for late talkers by examining its impact on both expressive lexical and syntactic skills and extending its implementation for the first time to a preterm population that is at higher risk for language delay than the general population. Overall, our findings suggested that a parent-implemented program based on dialogic book reading may reduce expressive lexical and syntactic delays in late talkers. However, population characteristics must also be considered for a deeper understanding of its effectiveness. Clinical implications of these results are discussed.

### 4.1. The Efficacy of the Parent-Implemented Intervention in Late Talkers

With respect to the first objective of the present study, our results revealed that a significantly higher rate of late talkers in the intervention group (63%) showed partial or total recovery from expressive lexical delay than children in the control group (35%). This result was consistent with the findings by Buschmann and colleagues [25], who found that after participating in a highly structured shared book reading intervention administered by parents, children in the treatment group made greater gains in their lexical skills than their peers in the control group. In addition, our results showed that 63% of children in the treatment group versus 22% of children in the control group acquired the ability to produce complete sentences. This is in line with evidence of previous studies [24,25], highlighting an increase in the production of multiword and structurally complete sentences of children with expressive lexical delay who receive intervention. Overall, this study suggests that indirect treatment is preferable to a “wait and see approach” in children with expressive language delay [22]. Indeed, after participating in parent-implemented intervention, a lower rate of children with expressive language delay may need direct individual treatment. Despite these encouraging results, our findings reveal that when considering the total sample, children in the intervention group did not show significant differences in daily growth rates of their expressive vocabulary size, its semantic composition, and complete and total sentence production with respect to their peers in the control group. These findings are not in line with previous studies that found a significant increase in expressive vocabulary in children who received the intervention [24,25]. The differences with our results may have several explanations. First, as described in the introduction, the aforementioned studies used a more rigorous study design, with a randomized assignment of children to the intervention and control groups. Therefore, we cannot exclude that the different results for children’s outcomes might at least partially depend on type of study design [20]. Second, we used less stringent inclusion criteria than those used in previous studies [24,25], since we included children with a wider range of cognitive skills excluding only children with an IQ below 70. The choice to include children with a wider intellectual quotient was motivated by evidence in the literature suggesting that late talkers are characterized by a wide heterogeneity of cognitive skills [10,41,42,43]. Third, previous studies included only late talkers without biological risk, whereas the present study included both full-term and low-risk preterm late talkers. The latter group is characterized by biological vulnerabilities that can affect language development (see Section 4.2 for the discussion on this point). Fourth, duration of the interventions adopted in previous studies differed from the intervention program used in this study. Indeed, parent-implemented interventions described by Buschmann and colleagues [25] and by Girolametto and colleagues [24] were longer (i.e., 12 weeks).

### 4.2. The Efficacy of Parent-Implemented Intervention in Low-Risk Preterm and Full-Term Late Talkers 

The second objective of our study was to investigate the impact of this parent-implemented language intervention on late talkers with vulnerabilities associated with specific biological conditions (i.e., low-risk preterm late talkers, compared to full-term late talkers). To our knowledge, no studies on the effectiveness of parent-implemented interventions have been carried out on preterm children. Only one study showed that perinatal risk (i.e., being admitted at the neonatal intensive care unit after birth) did not affect language outcomes of children participating in an intervention promoting language development [29]. However, in this latter study, the authors did not specify the neonatal birth status of the children as well as their gestational age. Therefore, the present study explored for the first time the effect of a parent-implemented program on expressive lexical and syntactic outcomes in function of preterm risk status.

Our findings revealed that after the intervention, in both the low-risk preterm and the full-term samples, a higher rate of children acquired the ability to produce complete sentences from the pre- to the post-intervention assessment with respect to children in the control group. In particular, regarding low-risk preterm late talkers, 53% of those participating in the intervention showed this acquisition, whereas none of the low-risk preterm children in the control group showed this new achievement. Regarding full-term late talkers, 80% of them acquired the ability to produce complete sentences at the post-intervention assessment versus 30% of the children in the control group. Nonetheless, the effects of the intervention were more evident in full-term late talkers, who showed a higher daily growth rate of total words, nouns, function words, and complete sentences than full-term late talkers in the control group.

When we consider only full-term late talkers, our results confirm previous findings of similar intervention programs [24,25] and expand them, specifying the rate of children not only recovering their expressive lexical delay but also mastering the production of complete sentences. In this sense, our evidence confirms that a parent-implemented intervention may be effective in improving both expressive lexical and syntactic skills of late talkers [24,25]. In addition, the current study provides new insights by examining change within semantic categories. As suggested in the literature, social words and nouns are the first lexical semantic categories acquired in typically developing children, whereas predicates and function words become more frequent when vocabulary exceeds 200 words [44,45]. Our findings supported this evidence, highlighting, in addition, that expressive language growth might be accelerated by an early parent-implemented intervention. 

Concerning low-risk preterm late talkers, our evidence suggests that a parent-implemented language intervention impacts on the emergent ability to produce multiword sentences. This result is consistent with what emerged in a review of studies involving another population at risk for language delay, i.e., children with developmental delay [28], and it highlights the need of further investigation of how biological vulnerabilities, such as preterm birth, could moderate the effect of a parent-based language intervention.

### 4.3. Limitations and Strengths of the Study 

The findings of this study must be considered in light of some limitations. First, in the present study, our control group is recruited with a convenience method, since our sample size is not large enough to randomly assign children to intervention and control groups. The randomized controlled design is considered the more rigorous design methodology and the golden standard for evaluating the efficacy of interventions [20,46]. In our study, the non-random assignment of participants to groups entailed some pre-existing differences between the intervention and the control groups concerning parental age and children’s production of social words, which was slightly higher in the intervention group. Given the small sample size, these variables were not controlled in the study’s main analyses, weakening the strength of our conclusions. Thus, further studies on the efficacy of parent-implemented intervention including larger late-talker samples and adopting a randomized controlled design and inserting covariates to control sources of variability on the effect of the intervention between groups should be conducted for late talkers born preterm and full-term. 

Second, we include low-risk preterm late talkers who usually do not have severe perinatal risks, limiting the generalization of our results to a subgroup of preterm children. Future studies should be conducted, including a sample of toddlers born preterm with a wider range of gestational ages. Third, the small number of low-risk preterm children who did not participate in the intervention does not allow for solid conclusions to be drawn about the effects of a parent-implemented intervention on the preterm population. Further studies should confirm and broaden the considerations that emerged from our study. Fourth, we examine children’s language outcomes only through parental reported measures. These measures have been used in previous studies [24,25,36,47], and no significant differences in effect sizes between parental reported measures and observational measures were described [23]. However, we cannot exclude a bias effect related to parental expectations. Therefore, multiple measures in pre- and post-intervention assessments should be used in future studies to examine intervention effects. Finally, in the present study, we focus only on children’s language outcomes. Since other studies on parent-implemented interventions highlighted changes in parents’ outcomes, in terms of their responsiveness, rate of communication, and language modeling [20,23], parental outcomes should also be investigated to understand how improvements in child communicative linguistic skills are associated to changes in parental communicative behaviors.

Nonetheless, the present study has several strengths. The main strength is exploring, for the first time, the effects of a parent-implemented intervention on both low-risk preterm and full-term late talkers. Our study reveals the effectiveness of a parent-implemented program based on dialogic book reading in populations with biological vulnerabilities, even if with some differences in the type of impact. A second strength is describing the change in children’s language outcomes after the intervention not only in global language outcomes, such as expressive vocabulary size, but also in more specific linguistic measures such as semantic categories of expressive vocabulary and production of complete sentences.

### 4.4. Implications for Practice 

The implications for practice are twofold. The first concerns the relevance of implementing early parent-based intervention for supporting children with expressive language delay. As described in the introduction, wide interindividual variability characterizes language development until three years of age [7,33]. In light of this, a widespread approach adopted by several clinicians and pediatricians is to “wait and see” and begin language intervention by preschool age. In contrast, our results show that supporting parents in using appropriate strategies to promote their child language development before three years of age could potentially change children’s short-term language progress [22]. In turn, this change may have positive cascading effects on their parents who may feel themselves more empowered to manage their child’s language difficulties, and on clinical services, which may reduce their waiting lists for treatment.

The second implication concerns the impact of the parent-implemented program. Our results reveal that an ecological program, in which parents are coached to be child-oriented, promoting interaction and using specific language modeling techniques in the context of dialogic book reading, may be effective in improving expressive lexical and syntactic skills of late talkers. In addition, the program provides a favorable cost–benefit ratio, being effective with a relatively short duration (about two months). The program appears effective in improving early syntactic skills of low-risk preterm late talkers, highlighting that biological vulnerabilities should be considered in evaluating the effect of the intervention in this population. Further studies on the most optimal strategies for supporting language development in the preterm population should be pursued.

## 5. Conclusions

The present study provides new evidence on the effectiveness of parent-implemented language interventions for late talkers by adopting a short, ecological, and cost-effective program. Overall, our findings highlight that a parent program using dialogic book reading enhances expressive lexical and syntactic skills of late talkers, helping them to fully or partially recover their expressive lexical delay as well as increase their ability to produce complete sentences. In addition, our findings reveal that the effects of this parent-based intervention are more evident in late talkers without biological vulnerabilities. Indeed, whereas full-term late talkers show gains in expressive vocabulary, its semantic categories and expressive syntactic skills, only significant gains in expressive syntactic skills were shown in low-risk preterm late talkers. This suggests the need to implement targeted interventions for specific populations of late talkers and promote evidence-based practices for supporting language development in the preterm population.

## Figures and Tables

**Table 1:** Sociodemographic characteristics of participants in the entire sample.

Participants’ Characteristics	Intervention	Control	χ^2^/*t* (df)	*p*
	(*n* = 27)	(*n* = 23)		
Gestational Age (weeks), Mean (SD)	36.29 (3.39)	37.81 (2.86)	1.70 (48)	0.096
Birthweight (grams), Mean (SD)	2464.37 (888.11)	2915.26 (696.13)	1.97 (48)	0.054
Length of Stay in Hospital (days), Mean (SD)	15.96 (33.92)	5.09 (6.50)	−1.51 (48)	0.137
Gender (Female), *n* (%)	10 (37.0)	7 (30.4)	0.24 (1, 50)	0.623
Firstborn, *n* (%)	14 (51.8)	9 (39.1)	2.09 (1, 50)	0.393
Twins, *n* (%)	9 (33.3)	5 (21.7)	0.83 (1, 50)	0.363
Otitis Media, *n* (%)	1 (3.7)	2 (8.7)	0.55 (1, 50)	0.459
Family History of Language and/or Learning Disorders (LLD), *n* (%)	6 (22.2)	4 (17.4)	0.18 (1, 50)	0.670
Nursery School Attendance, *n* (%)	23 (85.2)	15 (65.2)	2.72 (1, 50)	0.099
Other Parent Input Besides Italian, *n* (%)	6 (22.2)	1 (4.3)	3.30 (1, 50)	0.107
Mother’s Age (years), Mean (SD)	40.67 (4.82)	36.26 (4.80)	−3.23 (48)	**0.002**
Father’s Age (years), Mean (SD)	42.67 (4.98)	39.14 (5.82)	−2.19 (48)	**0.034**
Mothers with High Educational Level (>13 years), *n* (%)	17 (63.0)	13 (56.5)	1.23 (1, 50)	0.767
Fathers with High Educational Level (>13 years), *n* (%)	12 (44.4)	9 (39.1)	1.06 (1, 50)	0.601
Mother’s Nationality (Italian), *n* (%)	22 (81.5)	22 (95.6)	2.36 (1, 50)	0.124
Father’s Nationality (Italian), *n* (%)	23 (85.2)	22 (95.6)	1.51 (1, 50)	0.219
Age at Pre-Intervention (months), Mean (SD)	31.09 (1.06)	31.34 (1.02)	0.87 (48)	0.389
Age at Post-Intervention (months), Mean (SD)	36.72 (1.38)	37.18 (1.12)	1.27 (48)	0.210
Pre and Post-Intervention Interval (days), Mean (SD)	173.48 (34.96)	177.26 (39.90)	0.35 (48)	0.723

Significant results are displayed in bold.

**Table 2:** Sociodemographic characteristics of participants within the low-risk preterm and full-term samples.

Participants’ Characteristics	Low-Risk Preterm Children				Full-Term Children			
	Intervention	Control	χ^2^/*t* (df)	*p*	Intervention	Control	χ^2^/*t* (df)	*p*
	(*n* = 17)	(*n* = 6)			(*n* = 10)	(*n* = 17)		
Gestational Age (weeks), Mean (SD)	34.23 (2.30)	33.59 (1.73)	−0.61 (21)	0.548	39.80 (1.46)	39.30 (1.12)	−0.99 (25)	0.329
Birthweight (grams), Mean (SD)	1876.65 (468.12)	2031.17 (254.60)	0.76 (21)	0.455	3463.50 (356.17)	3227.29 (499.50)	−1.31 (25)	0.203
Length of Stay in Hospital (days), Mean (SD)	23.88 (41.09)	11.17 (10.81)	−0.74 (21)	0.468	2.50 (1.78)	2.94 (0.41)	0.65 (25)	0.524
Gender (Female), *n* (%)	4 (23.5)	2 (33.3)	0.22 (1, 23)	0.632	6 (60.0)	5 (29.4)	2.44 (1, 27)	0.224
Firstborn, *n* (%)	10 (58.8)	1 (16.7)	3.44 (1, 23)	0.171	4 (40.0)	8 (47.1)	1.69 (1, 27)	0.636
Twins, *n* (%)	9 (52.9)	5 (83.3)	1.72 (1, 23)	0.340	0 (0.0)	0 (0.0)	-	-
Otitis Media, *n* (%)	1 (5.9)	0 (0.0)	0.37 (1, 23)	1.000	0 (0.0)	2 (11.8)	1.27 (1, 27)	0.516
Family History of Language and/or Learning Disorders (LLD), *n* (%)	2 (11.8)	2 (33.3)	1.44 (1, 23)	0.231	4 (40.0)	2 (11.8)	2.90 (1, 27)	0.153
Nursery School Attendance, *n* (%)	14 (82.4)	2 (33.3)	5.03 (1, 23)	**0.045**	9 (90.0)	13 (76.5)	0.76 (1, 27)	0.621
Other Parent Input Besides Italian, *n* (%)	4 (23.5)	0 (0.0)	1.71 (1, 23)	0.539	2 (20.0)	1 (5.9)	1.27 (1, 27)	0.535
Mother’s Age (years), Mean (SD)	41.41 (5.50)	36.67 (3.20)	−2.12 (21)	**0.046**	39.40 (4.25)	36.12 (5.32)	−1.66 (25)	0.110
Father’s Age (years), Mean (SD)	43.33 (5.02)	40.60 (5.50)	−1.08 (19)	0.293	41.13 (4.85)	38.69 (6.01)	−0.99 (22)	0.331
Mothers with High Educational Level (>13 years), *n* (%)	8 (47.1)	4 (66.7)	0.68 (1, 23)	0.640	9 (90.0)	9 (52.9)	3.72 (1, 27)	0.133
Fathers with High Educational Level (>13 years), *n* (%)	5 (29.4)	2 (33.3)	3.07 (1, 23)	0.246	7 (70.0)	7 (41.2)	2.20 (1, 27)	0.389
Mother’s Nationality (Italian), *n* (%)	13 (76.5)	6 (100.0)	1.71 (1, 23)	0.539	1 (10.0)	16 (94.1)	0.16 (1, 27)	1.000
Father’s Nationality (Italian), *n* (%)	14 (82.4)	6 (100.0)	1.22 (1, 23)	0.539	1 (10.0)	16 (94.1)	0.16 (1, 27)	1.000
Age at Pre-Intervention (months), Mean (SD)	30.87 (0.66)	31.94 (0.91)	3.08 (21)	**0.006**	31.45 (1.50)	31.13 (1.00)	−0.65 (25)	0.520
Age at Post-Intervention (months), Mean (SD)	36.32 (1.31)	37.13 (1.41)	1.27 (21)	0.219	37.40 (1.29)	37.20 (1.05)	−0.44 (25)	0.661
Pre and Post-Intervention Interval (days), Mean (SD)	168.94 (29.61)	156.50 (38.17)	−0.82 (21)	0.419	181.20 (43.21)	184.59 (38.93)	−0.21 (25)	0.836

Significant results are displayed in bold.

**Table 3:** Cognitive and language skills of children at the pre-intervention assessment.

	All Children (*n* = 50)				Low-Risk Preterm Children (*n* = 23)				Full-Term Children (*n* = 27)			
	Intervention (*n* = 27)	Control (*n* = 23)			Intervention (*n* = 17)	Control (*n* = 6)			Intervention (*n* = 10)	Control (*n* = 17)		
	*M (SD)*	*M (SD)*	*U*	*p*	*M (SD)*	*M (SD)*	*U*	*p*	*M (SD)*	*M (SD)*	*U*	*p*
*MB-CDI Language Measures*												
Word Production	139.37 (106.90)	102.61 (97.70)	235.00	0.142	145.82 (122.09)	133.83 (64.35)	49.00	0.919	128.40 (79.38)	91.60 (106.47)	56.50	0.155
Social Words	30.33 (9.61)	23.35 (11.24)	201.50	**0.034**	29.59 (10.09)	28.83 (5.81)	50.00	0.973	31.60 (9.09)	21.41 (12.16)	44.00	**0.040**
Nouns	77.22 (66.21)	58.74 (65.78)	260.00	0.325	83.88 (75.12)	79.83 (51.57)	48.00	0.865	65.90 (49.03)	51.29 (69.96)	63.50	0.286
Predicates	19.04 (25.60)	12.57 (16.56)	252.50	0.256	20.59 (31.00)	16.50 (13.03)	41.50	0.516	16.40 (13.18)	11.18 (17.78)	53.50	0.115
Function Words	7.67 (6.72)	5.35 (6.38)	221.00	0.080	7.06 (6.67)	5.17 (3.97)	45.00	0.708	8.70 (7.04)	5.41 (7.14)	53.00	0.115
Incomplete Sentences	7.78 (11.73)	4.78 (7.03)	290.00	0.672	5.76 (9.62)	6.67 (11.65)	46.50	0.759	11.20 (14.57)	4.12 (4.86)	64.50	0.309
Complete Sentences	0.33 (1.21)	1.22 (3.34)	264.00	0.176	0.47 (1.50)	1.50 (2.59)	25.50	0.074	0.10 (0.32)	1.12 (3.71)	82.50	0.902
Total Sentences	8.11 (12.08)	6.00 (8.54)	301.00	0.844	6.24 (10.41)	8.17 (11.79)	42.00	0.562	11.30 (14.52)	5.24 (7.39)	66.50	0.359
*Bayley-III Cognitive Measure*												
Cognitive Composite Score	90.93 (10.10)	85.65 (8.57)	216.50	0.064	89.12 (10.34)	84.17 (7.36)	37.00	0.354	94.00 (9.37)	86.17 (9.10)	46.50	0.052

Significant results are highlighted in bold.

**Table 4:** Children’s change from pre- to post-intervention in expressive lexical delay and expressive syntactic skills.

	All Children (*n* = 50)				Low-Risk Preterm Children (*n* = 23)			Full-Term Children (*n* = 27)		
Children’s Change from Pre- to Post-Intervention	Intervention	Control			Intervention	Control		Intervention	Control	
	(*n* = 27)	(*n* = 23)	*χ* ^2^	*p*	(*n* = 17)	(*n* = 6)	Fisher’s *p*	(*n* = 10)	(*n* = 17)	Fisher’s *p*
*Expressive lexical delay*			3.94	**0.047**			0.371			0.120
Children partially or fully recovering, *n* (%)	17 (63)	8 (35)			10 (59)	2 (33)		7 (70)	6 (35)	
Children not recovering, *n* (%)	10 (37)	15 (65)			7 (41)	4 (67)		3 (30)	11 (65)	
*Expressive syntactic skills*			8.60	**0.013**			**0.010**			**0.018**
Children with stable complete sentences, *n* (%)	3 (11)	6 (26)			2 (12)	4 (67)		1 (10)	2 (12)	
Children with emergent complete sentences, *n* (%)	17 (63)	5 (22)			9 (53)	0 (0)		8 (80)	5 (30)	
Children with stable incomplete sentences, *n* (%)	7 (26)	12 (52)			6 (35)	2 (33)		1 (10)	10 (59)	

Significant results are highlighted in bold.

**Table 5:** Children’s daily growth rate in expressive lexical and syntactic skills from pre- to post-intervention assessment.

Daily Growth Rate	All Children (*n* = 50)				Low-Risk Preterm Children (*n* = 23)				Full-Term Children (*n* = 27)			
	Intervention	Control			Intervention	Control			Intervention	Control		
	(*n* = 27)	(*n* = 23)	U	*p*	(*n* = 17)	(*n* = 6)	U	*p*	(*n* = 10)	(*n* = 17)	U	*p*
Total words	1.20 (0.77)	0.84 (0.88)	225.00	0.096	0.95 (0.62)	0.94 (0.90)	50.00	0.944	1.61 (0.85)	0.81 (0.90)	44.00	**0.040**
Social words	0.07 (0.05)	0.08 (0.08)	271.00	0.442	0.07 (0.03)	0.06 (0.10)	37.00	0.327	0.07 (0.06)	0.08 (0.07)	85.00	1.000
Nouns	0.67 (0.43)	0.45 (0.48)	219.00	0.075	0.57 (0.42)	0.53 (0.49)	46.00	0.795	0.82 (0.43)	0.42 (0.49)	44.00	**0.040**
Predicates	0.29 (0.25)	0.22 (0.26)	250.00	0.239	0.21 (0.16)	0.23 (0.23)	46.00	0.726	0.44 (0.30)	0.22 (0.28)	49.00	0.071
Function words	0.09 (0.07)	0.06 (0.06)	222.50	0.087	0.06 (0.07)	0.07 (0.06)	49.00	0.889	0.14 (0.06)	0.05 (0.07)	30.00	**0.005**
Complete sentences	0.06 (0.07)	0.03 (0.06)	227.00	0.093	0.04 (0.07)	0.04 (0.07)	44.00	0.617	0.09 (0.08)	0.03 (0.06)	45.00	**0.046**
Total sentences	0.12 (0.09)	0.09 (0.09)	241.00	0.175	0.13 (0.10)	0.13 (0.12)	50.50	0.973	0.10 (0.08)	0.07 (0.07)	65.00	0.334

Significant results are highlighted in bold.

## Appendix A: Clinical and Perinatal Characteristics of Low-Risk Preterm Children

**Table A1:** Clinical and Perinatal Characteristics of Low-Risk Preterm Children.

Clinical and Perinatal Characteristics	Low-Risk Preterm Children (*n* = 23)
	*n* (%)
Caesarean Section	22 (95.65)
SGA	6 (26.08)
IVH I/II	0 (0)
MV	1 (4.35)
RDS	12 (52.17)
Apnea	1 (4.35)
BDP	0 (0)
Sepsis	1 (4.35)
ROP I/II	0 (0)
Hyperbilirubinemia with Phototherapy, *n* (%)	15 (65.22)

SGA: Small for gestational age, infants with a birthweight < 10th percentile for gestational age; IVH I/II: intra-ventricular hemorrhage originating within the subependymal germinal matrix filling less than 10% (I grade) and 50% (II grade), respectively, of the ventricular area on parasagittal view; MV: mechanical ventilation; RDS: respiratory distress syndrome, acute illness coming on within 4–6 h of delivery, characterized clinically by respiratory rate ≥ 60/min, dyspnea and respiratory distress; Apnea: more than four episodes of apnea/hour or more than two episodes of apnea/hour if ventilation with a bag and mask was required; BPD: bronchopulmonary dysplasia, needing both supplemental oxygen for ≥ 28 days and at 36 weeks of post-conception age; Sepsis: presence of a positive blood culture and/or clinical and laboratory signs; ROP I/II: retinopathy of prematurity, vasoproliferative retinopathy resolved without a specific therapy before the presumed date of birth; Hyperbilirubinemia with phototherapy: hyperbilirubinemia needing phototherapy according to the criteria proposed by Gomella [48].
